# Melatonin Suppresses the Growth of Ovarian Cancer Cell Lines (OVCAR-429 and PA-1) and Potentiates the Effect of G1 Arrest by Targeting CDKs

**DOI:** 10.3390/ijms17020176

**Published:** 2016-01-29

**Authors:** Ching-Ju Shen, Chi-Chang Chang, Yi-Tz Chen, Chung-Sheng Lai, Yi-Chiang Hsu

**Affiliations:** 1 Graduate Institute of Medicine, College of Medicine, Kaohsiung Medical University, 807 Kaohsiung, Taiwan; chenmed.tw@yahoo.com.tw; 2 Department of Gynecology and Obstetrics, Kaohsiung Medical University Hospital, Kaohsiung Medical University, 807 Kaohsiung, Taiwan; 3 Department of Obstetrics & Gynecology, E-Da Hospital, E-Da Hospital/I-Shou University, 82445 Kaohsiung, Taiwan; p2696373@yahoo.com.tw; 4 Graduate Institute of Medical Science, College of Health Sciences, Chang Jung Christian University, 71101 Tainan, Taiwan; amy073640171@yahoo.com.tw; 5 Innovative Research Center of Medicine, College of Health Sciences, Chang Jung Christian University, 71101 Tainan, Taiwan; 6 Division of Plastic Surgery, Kaohsiung Medical University Hospital, Kaohsiung Medical University, 807 Kaohsiung, Taiwan

**Keywords:** ovarian cancer, melatonin, cell cycle, CDK

## Abstract

Melatonin is found in animals as well as plants. In animals, it is a hormone that anticipates the daily onset of darkness and regulates physiological functions, such as sleep timing, blood pressure, and reproduction. Melatonin has also been found to have anti-tumor properties. Malignant cancers are the most common cause of death, and the mortality rate of ovarian tumor is the highest among gynecological diseases. This study investigated the anti-tumor effects of melatonin on the ovarian cancer lines, OVCAR-429 and PA-1. We observed the accumulation of melatonin-treated cells in the G_1_ phase due to the down-regulation of CDK 2 and 4. Our results suggest that in addition to the known effects on prevention, melatonin may also provide anti-tumor activity in established ovarian cancer.

## 1. Introduction

In 2014, there were nine major advances in clinical research on gynecologic oncology: two each in cervical and corpus cancer, four in ovarian cancer, and one in breast cancer [1]. Improvements in ovarian cancer therapies have allowed a greater number of young women to survive; however, many of these patients suffer from ovarian failure or early menopause followed by the loss of reproductive function [2]. Cryopreservation is used to preserve fertility in young patients undergoing chemotherapy or radiation; however, this option is not accessible to all cancer patients [3]. PARP inhibitors, olaparib (AZD2281) [4,5], and rucaparib (CO338), have been undergoing clinical trials [1] since they were first shown to have anti-tumor activity in BRCA-related ovarian cancer [4].

Melatonin is synthesized from the amino acid tryptophan via conversion to serotonin and *N*-acetylserotonin is converted into melatonin by the enzyme hydroxyindole-*O*-methyltransferase (HIOMT) [6]. Pineal melatonin production exhibits a circadian rhythm, with low levels expressed during the day and higher levels at night [7]. Melatonin is derived almost entirely from the pineal gland, as shown by the fact that undetectable melatonin levels are found [8]. After its release, melatonin is bound to albumin and reaches tissues within a very short period [9]. The melatonin molecule possesses lipophilic and hydrophilic properties that permit its transfer into many tissues and fluids. Depending upon the site of production and target organ, melatonin can act as a hormone, immuno-modulator or as a biological modifier [10]. Melatonin has been identified as a natural chronobiotic substance with immune-enhancing properties as well as the ability to reduce oxidative stress. Thus, melatonin could potentially be used as a therapeutic substance for the prevention or arrest of neoplastic growth [11]. The concentrations of melatonin in ovarian follicles tend to increase with follicular growth [12]. It is possible that melatonin acts as an antioxidant [13]. Furthermore, the anti-apoptotic effect of melatonin has been observed in a variety of cell types [14]. And there are indications that melatonin can help prevent and treat oxidative damage as well as suppress specific inflammatory factors [15].

The aim of this study was to investigate the protective effects of melatonin on anti-proliferation and cell cycle regulation in ovarian cancer cells (OVCAR-429 and PA-1). We performed these experiments with the aim of providing technological support for the further development of therapies for ovarian cancer.

## 2. Results and Discussion

### 2.1. Melatonin Can Mediate the Survival of Ovarian Cancer Cells and Thereby Inhibit Proliferation

**Figure 1:**
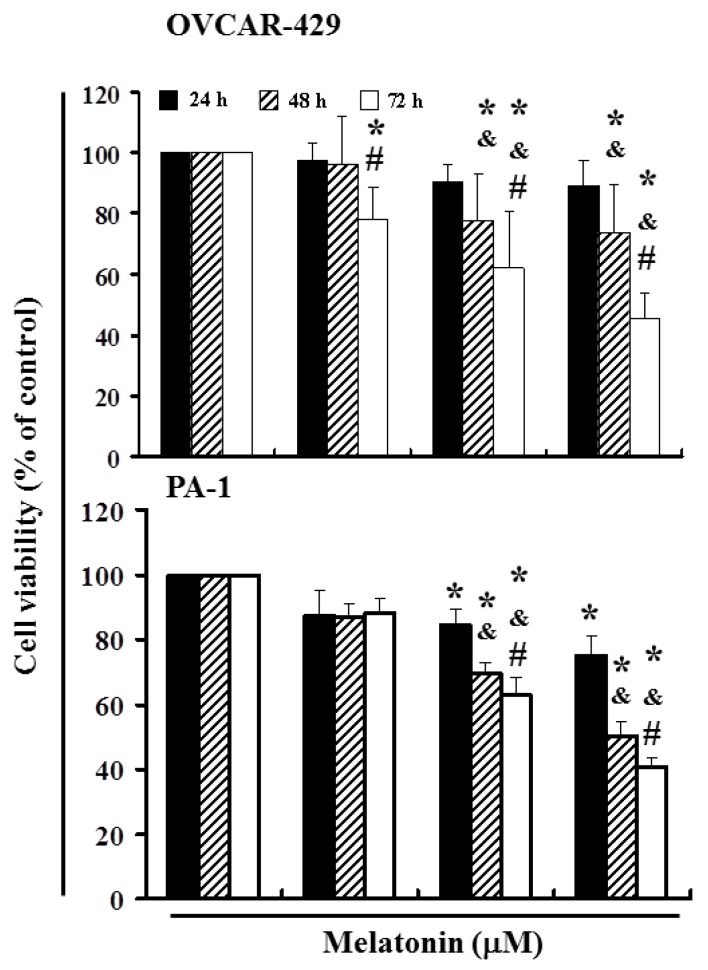
Melatonin mediates the cell viability of ovarian cancer cell lines (OVCAR-429 and PA-1), thereby inhibiting proliferation. An *in vitro* study was initiated by treating each of the cancer cells with increasing doses of melatonin (0, 400, 600, and 800 µM) for 1 to 3 days. We determined the viability of melatonin-treated cancer cells using the MTT test. The results were expressed as a percentage of control group, which was considered 100%. All data were reported as the mean (±SEM) of at least 3 separate experiments. Statistical analysis significance was performed assessed using a *t*-test, with significant differences determined between experimental and control groups considered to be significant at the level of * *p* < 0.05 *versus* the control group, while the symbol on the bar denotes the difference is statistically significant at *p* < 0.05 as compared to the 24 h (^&^) or 48 h (^#^).

To explore this anti-tumor activity, we conducted an *in vitro* study in which OVCAR-429 and PA-1 cell lines were subjected to increasing dosages of melatonin (0, 400, 600, and 800 µM) for a period of between 24 and 72 h. We then measured the proliferation of melatonin-treated cancer cells by the MTT [3-(4,5-dimethylthiazol-2-yl)-2,5-diphenyltetrazolium bromide] test (Figure 1). The results indicate that melatonin treatment reduced the survival and proliferation of OVCAR-429 and PA-1 cell lines (Figure 1) (* *p* < 0.05 *versus* melatonin 0 µM) in a dose- and time-dependent manner.

### 2.2. Non-Melatonin-Induced Apoptosis/Necrosis of OVCAR-429 and PA-1 Cell Lines

**Figure 2:**
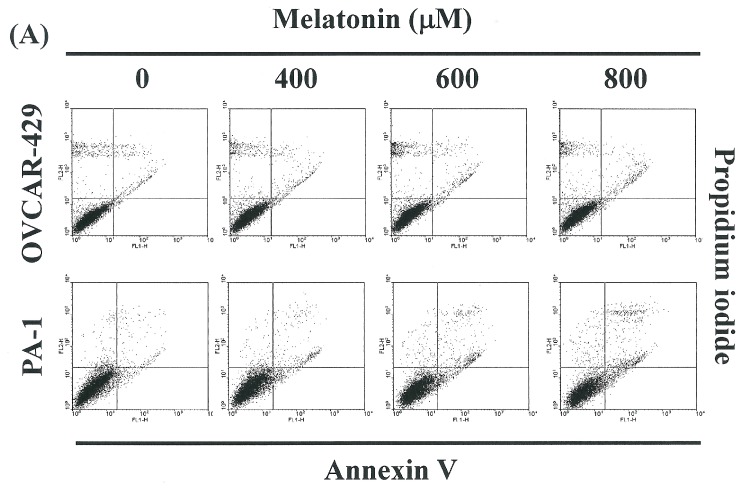

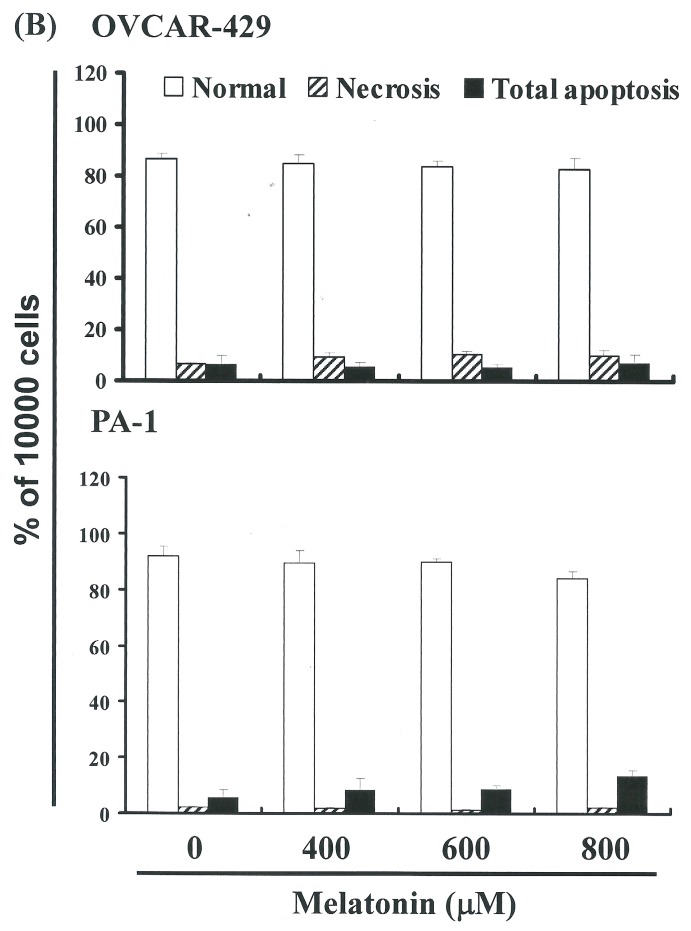
(**A**) the influence of melatonin on apoptosis and necrosis in OVCAR-429 and PA-1 cell lines; (**B**) Total apoptosis/necrosis in OVCAR-429 and PA-1 cells following incubation with melatonin for 4 h.

To identify the role played by melatonin in the apoptosis/necrosis of OVCAR-429 and PA-1 cells, we employed propidium iodide and annexin V-FITC staining to reveal the formation of apoptotic cells following treatment with melatonin for a period of 4 h. The percentage of apoptotic cells was assessed by flow cytometry (Figure 2A). A dot-plot of Annexin V-FITC fluorescence *versus* PI fluorescence indicates a non-significant increase in the percentage of apoptotic cells treated with melatonin, compared with untreated cells (melatonin 0 µM). No significant increase was observed in the percentage of cells undergoing necrosis, apoptosis (Figure 2B) or caspase 3 activation at melatonin concentrations of 400 to 800 μM (data not shown). Nonetheless, the results summarized in Figure 1 and Figure 2 indicate that melatonin may mediate the survival of OVCAR-429 and PA-1 cells. Thus, we hypothesize that pathways other than those associated with apoptosis and necrosis inhibited the proliferation of ovarian cancer cells.

### 2.3. Melatonin-Induced Accumulation of Melatonin-Treated Cells in the G_1_ Phase

**Figure 3:**
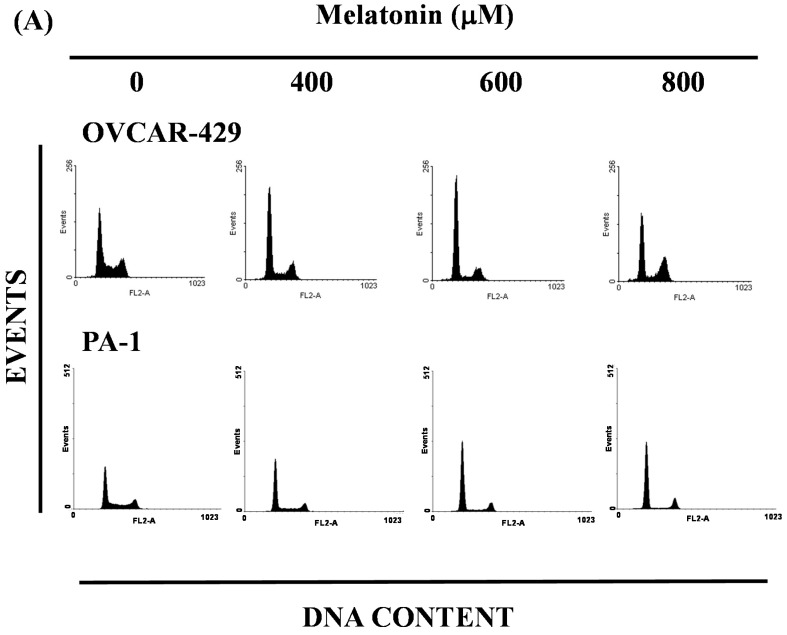

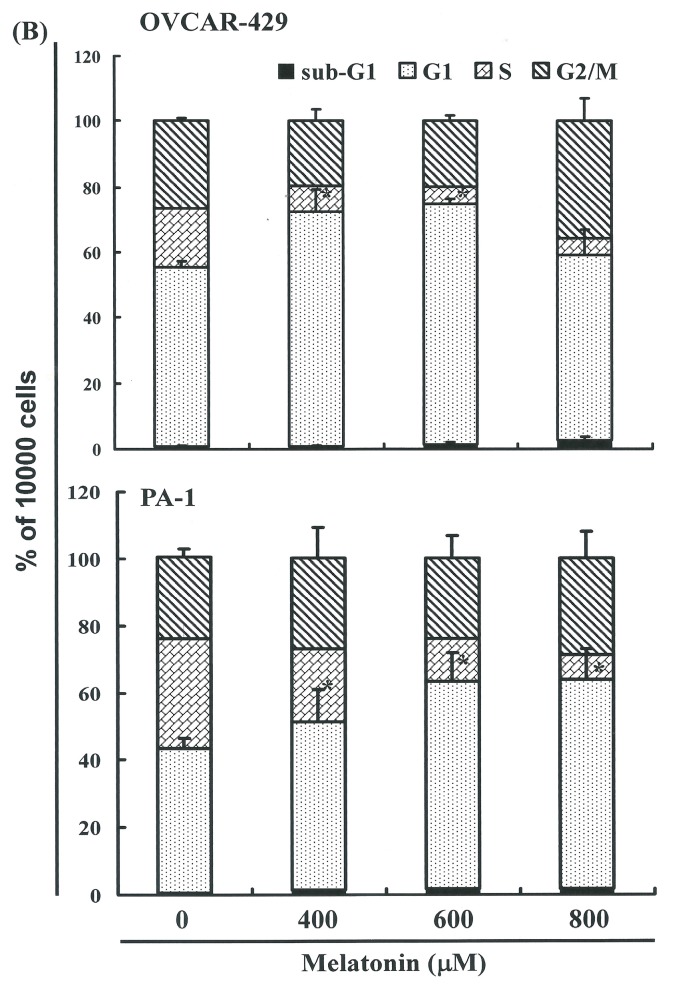
Influence of melatonin on cell cycle progression/distribution in OVCAR-429 and PA-1 cells: (**A**) Cell cycle analysis of ovarian cancer cell lines after being cultured with melatonin for 24 h; (**B**) melatonin induced an increase in G_1_ phase cells (%).The * symbol indicates that the difference resulting from treatment with melatonin 0 µM is statistically significant at *p* <0.05.

The cell-cycle (DNA) distribution of melatonin-treated cells was analyzed by flow cytometry. The cells were exposed to melatonin for one day prior to processing and analysis. As shown in Figure 3A, exposure to melatonin resulted in an increase in the number of cells in the cell cycle G_1_ phase, which implies that the OVCAR-429 and PA-1 cell lines underwent cell cycle arrest. Our results indicate that melatonin treatment increased the number of cells in the G1 phase, while simultaneously decreasing the number of cells in the S phases (* *p* < 0.05 *versus* melatonin 0 µM), but increasing the G_2_/M and subG1 in 800 µM melatonin treatment. (Figure 3B). Martín-Renedo *et al.* [16] also found the melatonin induced cell cycle arrest and apoptosis in hepatoma cells.

**Figure 4:**
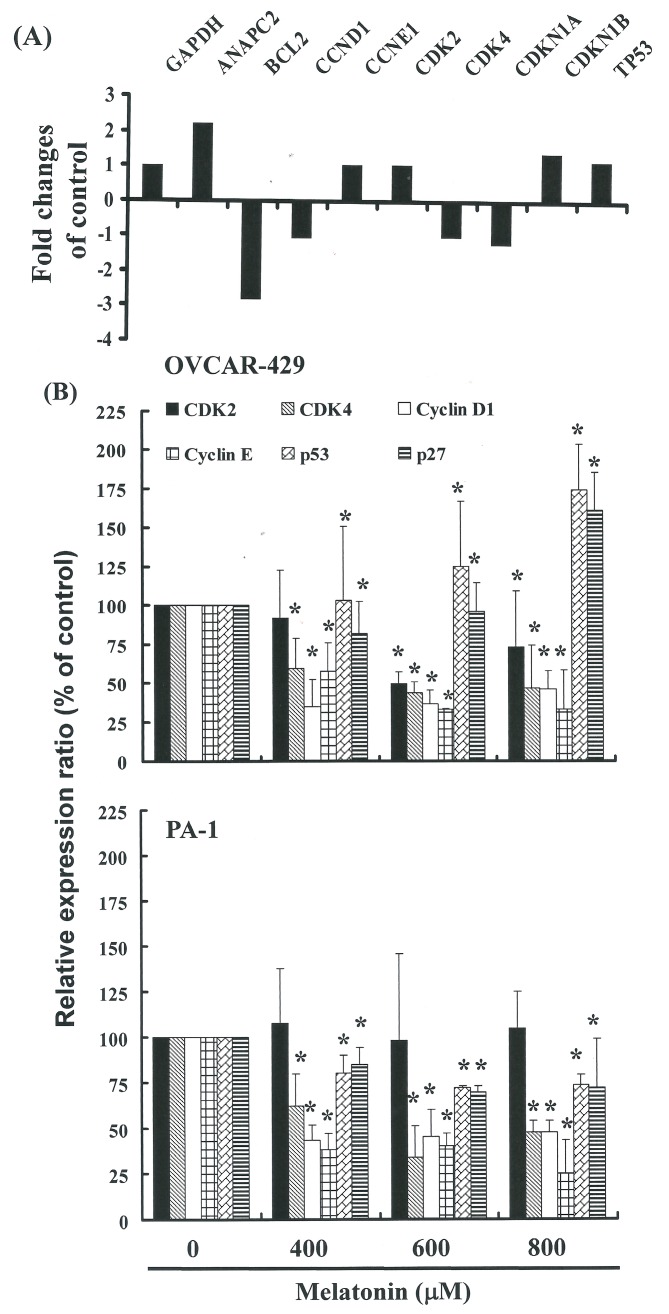
(**A**) The focused panel gene expression profile of OVCAR-429 cells was studied using the RT^2^ Profilter^TM^ PCR array following 4 h-exposure to vehicle (DMSO) or melatonin. Fold changes greater than 2 (up-regulation) or smaller than 0.5 (down-regulation) were considered substantial. Fold changes were calculated by dividing expression levels in melatonin-treated groups by expression levels in the vehicle-treated cells. ANAPC2: Anaphase promoting complex subunit 2, BCL2: B-cell CLL/lymphoma 2, CCND1: Cyclin D1, CCNE1: Cyclin E1, CDK2; Cyclin-dependent kinase 2, CDK4: Cyclin-dependent kinase 4, CDKN1A: Cyclin-dependent kinase inhibitor 1A (p21, Cip1), CDKN1B: Cyclin-dependent kinase inhibitor 1B (p27, Kip1), TP53: Tumor protein p53; (**B**) Melatonin induced a gene expression in OVCAR-429 and PA-1 cell lines. Quantitative RT-PCR (qPCR) analysis of CDK 2, 4, p27, and p53 mRNA expression standardized against the levels of GAPDH in ovarian cancer cell lines exposed for 4 h to DMSO (melatonin 0 µM control) or melatonin. Results were expressed as a percentage of control, which was considered 100%. All data were reported as the mean (±SEM) of at least three separate experiments. Symbol (*) in each group of bars indicates that the difference resulting from treatment with melatonin 0 µM is statistically significant at *p* < 0.05.

Principal component analysis (PCA) revealed in the PCR-array data derived from melatonin- and DMSO-treated cells. This suggests that treatment with melatonin had a far greater impact on the gene expression profile than could be reasonably attributed to technical errors. Therefore we divided the expression levels in the melatonin-treated group by those of the vehicle-treated group and considered changes more than 2-fold to be substantial up-regulation and changes smaller than 0.5-fold to be downregulation (Figure 4A). The findings indicate that common molecular pathways play roles in cell cycle regulation. The results of RT-PCR (Data not shown) and qPCR analysis (Figure 4B) were further validated using PCR-array analysis, which indicated substantial downregulation of CDKs (Figure 4A) as well as notable up-regulation of p27 and p53 mRNA expression in OVCAR-429 cells following exposure to melatonin (Figure 4B). These results indicate that melatonin may delay the growth of cancer cells in the G_1_ phase via down-regulation of CDK gene expression and/or the up-regulation of p53 and p27.

### 2.4. G_1_ Phase Arrest in Melatonin-Treated Cells via down Regulation of CDK2 and 4

**Figure 5:**
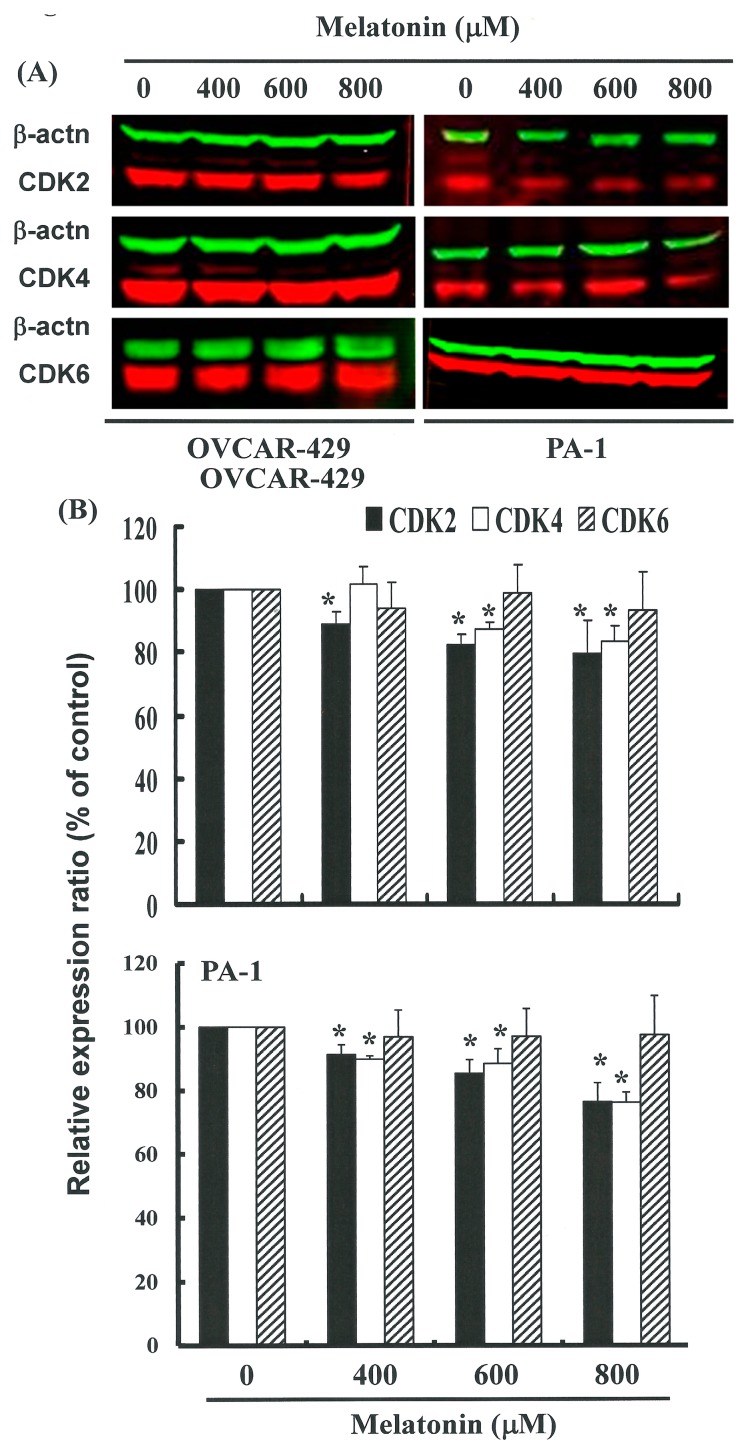
Melatonin represses CDKs gene expression in OVCAR-429 cells. (**A**) The cells were treated with melatonin (0, 400, 600, and 800 μM) for 24 h and gene expression of CDKs was subsequently detected by Western blot analysis; representative blot from three independent experiments; (**B**) Quantification of band intensities. Results are expressed as a percentage of the control sample, which was set at 100%. All data is presented as the mean (±SEM) of no fewer than three experiments. *t*-tests were used for all statistical analysis with significant differences determined by a *p* value of * <0.05 versus 0 μM control group.

Figure 4B illustrates mRNA gene expression and immunoblotting results (Figure 5A,B) of cellular proteins from OVCAR 429 and PA-1 cell lines treated with melatonin. Gene expression and Western blot analysis revealed a decrease in CDK2 and 4 following incubation with melatonin (Figure 4 and Figure 5). This confirms that CDK2 and 4 levels were significantly lower in cells incubated with melatonin.

Ovarian cancer is the leading cause of death due to gynecological malignancy. In 2012, there were an estimated 239,000 new cases of ovarian cancer worldwide, which would ultimately lead to more than 140,000 deaths [17]. Ovarian cancer produces few perceptible symptoms when localized to the ovary. Due to the asymptomatic nature of the disease in its early stages, most patients do not seek medical care until the disease has progressed beyond the ovaries into the abdomen and/or pelvis [18]. Novel therapies must be developed for integration into conventional treatment strategies in order to achieve improved clinical outcomes.

Low melatonin secretion levels have been reported in patients with endometrial cancer, but not in those with non-invasive ovarian cancer or squamous cervical cancer [19]. The exposure of BG-1 cells (ovarian adenocarcinoma cell line) to melatonin was shown to reduce the number of cells by 20% to 25% [20]. Immunofluorescence demonstrated MT1_CM_ expression in ovarian cell lines [21]. At concentrations of 0.001 nM–1 μM, melatonin did not exert anti-proliferative effects on ovarian cancer cells; however, it enhanced sensitivity to cisplatin [22]. Over the last two decades, advances in our understanding of ovarian cancer immunogenicity have opened the door to immunotherapeutic approaches to the treatment of ovarian cancer; for example, in the analysis of intraperitoneal (IP) immunotherapy in ovarian cancer [23]. The fact that melatonin increases the production of IL-2 means that melatonin could play a potential therapeutic role as a modulator of IL-2 production by T helper cells [24]. Melatonin could perhaps also be used as a novel oncostatic adjuvant agent [25]. Melatonin has been shown to inhibit tumor development under *in vivo* as well as *in vitro* conditions. There are five mechanisms by which melatonin could exert oncostatic effects: (a) via direct pro-apoptotic actions; (b) via antioxidant actions; (c) by reducing the uptake of key factors implicated in tumor growth signaling molecules; (d) by enhancing the immune system; (e) through anti-angiogenic effects [26]. Melatonin also activates various pathways involved in apoptosis and has been identified as a novel Sirt1 inhibitor in prostate cancer [27].

The results collected in this series of studies provide experimental evidence to support the contention that melatonin may irreversibly arrest the growth of OVCAR-429 cancer cells. The results of mechanistic analysis have led us to the conclusion that the inhibition of proliferation and the induction of cell cycle arrest are both strongly influenced by the accumulation of melatonin in cancer cells.

## 3. Experimental Section

### 3.1. Materials

MTT [3-(4,5-dimethylthiazol-2-yl)-2,5-diphenyltetrazolium bromide], Melatonin, and DMSO (dimethyl sulfoxide) were obtained from Sigma (St. Louis, MO, USA). Polyvinylidene fluoride membrane (PVDF) (Millipore, Merck KGaA, Darmstadt, Germany) and molecular weight marker were purchased from Bio Rad (Berkeley, CA, USA). Culture medium (minimum essential medium, RPMI 1640), fetal bovine serum (FBS), antibiotics, trypsin, sodium pyruvate, and phosphate-buffered saline (PBS) were purchased from Gibco, BRL (Grand Island, NY, USA). All other reagents and compounds were analytical grades.

### 3.2. Cells

The OVCAR-429 and PA-1 cell lines were from ATCC. The cells were maintained on culture dishes, in RPMI 1640 (OVCAR-429) or MEM (PA-1) supplemented with 10% (*v*/*v*) FBS. The cells were grown as monolayers in 95% humidified air with 5% CO_2_ at 37 °C.

### 3.3. Cell Proliferation Assay

The cells were seeded into 96-wells culture plate at 5000 cells/well. The cells were treated with 0, 400, 600, and 800 µM melatonin, the melatonin will complex with medium. Then the cell was incubated at 37 °C for 1 to 3 days in the CO_2_ incubator. After incubation of 1, 2 and 3 days the cells were treated with MTT dye (1 mg/mL) for at least 4 h. The reaction was stopped by DMSO, and the OD (optical density) was measured at 540 nm on a multi-wells plate reader. Background absorbance of the medium in the absence of cells was subtracted. All samples were assayed in triplicate, and the mean for each experiment was calculated. Results were expressed as a percentage of control, which was considered as 100%. Each assay was carried out in triplicate and the results were expressed as the mean (±SEM).

### 3.4. Measurement of Apoptosis

OVCAR-429 cells were first seeded in 6-well plates (Orange Scientific, Braine-l’Alleud, Belgium). Following treatment with melatonin for four hours, the cells were harvested. The cells were re-centrifuged (the supernatant discarded) and resuspended/incubated in 1× annexin-binding buffer, 5 μL of annexin V-FITC (BD Pharmingen, BD, USA) and 1 μL of 100 μg/mL PI working solution for 15 min. Following the incubation period, the stained cells were analyzed using flow cytometry (FACSCalibur, BD, USA). Data was analyzed using WinMDI 2.8 free software (BD, Franklin Lakes, NJ, USA).

### 3.5. Cell Cycle Analysis

For cell cycle analysis we used the fluorescent nucleic acid dye propidium iodide (PI) to identify the proportion of cells in each of the three interphase stages of the cell cycle. Cells were treated with melatonin for one day, and then harvested and fixed in 1 mL cold 70% alcohol for at least eight hours at −20 °C. DNA was stained in PI/RNaseA solution and the DNA content was detected using flow cytometry. Data was analyzed using WinMDI 2.8 free software.

### 3.6. Western Blot Assay

A total of 50 µg of proteins were separated by 10% SDS-PAGE, and transferred to PVDF membranes (Millipore, Merck KGaA, Darmstadt, Germany). The membranes were blocked with blocking buffer (Odyssey LI-COR, Lincoln, NE, USA) overnight, and incubated with anti-β-actin (Sigma-Aldrich, St. Louis, MO, USA), anti-CDK2, anti-CDK4, and anti-CDK6 (Santa Cruz BioTechnology, Dallas, TX, USA) antibodies for 1.5–2 h. The blots were washed and incubated with a second antibody (IRDye Li-COR, Lincoln, NE, USA) at a 1/20,000 dilution for 30 min. The antigen was then visualized using a near infrared imaging system (Odyssey LI-COR, Lincoln, NE, USA). The data was analyzed using Odyssey 2.1 software (Odyssey LI-COR, Lincoln, NE, USA).

### 3.7. Gene Expression Profiling (GEP)

Briefly, the cells untreated or treated with melatonin (600 µM) for 4 h, were harvested and total RNA was isolated utilizing an RNasey kit (Qiagen Taiwan, Taipei, Taiwan) as described by the manufacturer. The focused panel of genes was analyzed by RT² Profiler™ PCR Array (PAHS-020Z, Qiagen Taiwan, Taipei, Taiwan).

### 3.8. RT-PCR

A reverse transcriptase system (Promega, Southampon, UK) was used to synthesize cDNA from 1 µg of total RNA. Between 2 and 4 µL of cDNA were used for PCR analysis. PCR (50 µL) reactions were performed using 100 ng of each primer and 1 unit of Dynazyme II (Flowgen, Lichfield, UK). Thermal cycling was conducted for 35 cycles at the following temperature/durations: 98 °C for 10 s, 62 °C for 30 s, and 72 °C for 1 min using a Progene thermal cycler (Cambridge, UK). A final extension of 72 °C was performed for 10 min at the end of 35 cycles. The primers used for amplification of the target genes were checked against all other gene sequences for specificity. PCR reactions were analyzed on 1.5% agarose/TAE minigels and stained using 0.5 µg/mL ethidium bromide. Gels were visualized using an Apligene UV CCD camera system (Apligene, Heidelberg, Germany).

### 3.9. Real-Time PCR

Real-time PCR was conducted using SYBR Green PCR MasterMix (Carlsbad, CA, USA) according to the manufacturer’s instructions. Quantitative real-time PCR (qRT-PCR) was performed using approximately 200 ng of SYBR Green PCR MasterMix in an ABI 7300 system (Applied Biosystems, Foster City, CA, USA). PCR conditions were 95 °C for 120 s, 62 °C for 30 s, and 72 °C for 30 s for 40 cycles. Sample cells from three plates were run in duplicate, using the threshold suggested by the software for the instrument to calculate *C*_t_. To normalize readings, we used *C*_t_ values from 18 s as internal controls for each run, obtaining a delta *C*_t_ value for each gene.

### 3.10. Statistical Analysis

All data were reported as the mean (±SEM) of at least three separate experiments. A *t*-test or one-way ANOVA with *post-hoc* test was employed for statistical analysis, with significant differences determined as *p* < 0.05.

## 4. Conclusions

In conclusion, our findings highlight the role of melatonin in the inhibition of tumor growth through the delay of G_1_/S through the down-regulation of CDK2 and 4 in OVCAR-429 and PA-1 cell lines. This is the first study to demonstrate that the down-regulation of CDKs may at least partly explain the anti-cancer effects of melatonin against human ovarian cancer. This study provides one possible therapeutic strategy for the treatment of advanced ovarian cancer.
